# Cyclin G1 Regulates the Alveolarization in Models of Bronchopulmonary Dysplasia by Inhibiting AT2 Cell Proliferation

**DOI:** 10.3390/biom15010101

**Published:** 2025-01-10

**Authors:** Panpan Xu, Wanqing Zhuo, Peipei Zhang, Ying Chen, Yue Du, Ying Li, Yajuan Wang

**Affiliations:** 1 Children’s Hospital Capital Institute of Pediatrics, Chinese Academy of Medical Sciences & Peking Union Medical College, Beijing 100020, China; ey_xpp@student.pumc.edu.cn (P.X.);; 2 School of Life Sciences, Peking University, Beijing 100871, China; 3 Department of Neonatology, Children’s Hospital, Capital Institute of Pediatrics, Beijing 100020, Chinady623877932@163.com (Y.D.);

**Keywords:** bronchopulmonary dysplasia (BPD), type 2 alveolar (AT2) cells, cyclin G1 (CCNG1), Wnt signaling

## Abstract

Disrupted neonatal lung alveologenesis often leads to bronchopulmonary dysplasia (BPD), the most common chronic lung disease in children. The inhibition of type 2 alveolar (AT2) cell proliferation plays an important role in the arrest of alveologenesis. However, the mechanism of AT2 cell proliferation retardation in BPD is still not fully elucidated. The purpose of the present study was to explore the effects of cyclin G1 (CCNG1) on AT2 cell proliferation in hyperoxia-induced lung injury in neonatal mice. Our findings revealed that hyperoxia significantly reduced the proportion of AT2 cells in the lungs of neonatal mice and coincided with an upregulation of CCNG1 expression. Notably, this upregulation of CCNG1 was accompanied by an increase in Wnt signaling. We observed colocalization of CCNG1 and Wnt3a within AT2 cells in the hyperoxia group. Further analysis showed that inhibiting CCNG1 expression regressed the expression of Wnt signaling and enhanced cell proliferation. These results suggest that CCNG1 plays a pivotal role in suppressing AT2 cell proliferation, at least partly by counteracting the effects of Wnt signaling to modulate AT2 cell growth in the BPD model. Our findings contribute to a better understanding of the mechanisms underlying BPD.

## 1. Introduction

Bronchopulmonary dysplasia (BPD), predominantly affecting preterm infants with a gestational age of less than 32 weeks and a birth weight of under 1000 g, presents a rising incidence amidst advancements in perinatal medicine that have significantly boosted survival rates for premature births. The occurrence of BPD is closely related to premature lung exposure to hyperoxia, which leads to disrupted alveologenesis [1,2]. The mechanisms involved in the arrest of alveologenesis in response to hyperoxia remain unclear, despite decades of studies using rodent models [3,4]. Type 2 alveolar (AT2) cells are resident lung progenitor cells [5], which in neonatal lungs are more susceptible to hyperoxia, and a reduced number of AT2 cells leads to poor growth potential of BPD lungs [6]. Studies have shown that the proliferation of AT2 cells is significantly reduced in the BPD model induced by hyperoxia [7,8,9]. However, the mechanism of AT2 cell proliferation retardation in BPD is still not fully elucidated.

Wnt represents a family of highly conserved, secreted glycoproteins that play pivotal roles in cell proliferation, migration, polarity, and differentiation. Wnt proteins of classical Wnt signaling pathways primarily include Wnt1, Wnt2, and Wnt3a, and Wnt proteins of non-classical Wnt signaling pathways encompass Wnt4, Wnt5a, and Wnt6 [10]. Canonical Wnt signals are transmitted through Frizzled and LRP5/6. In the absence of Wnt signaling, intracellular β-catenin is captured by a specific protein complex (APC, Axin, and GSK-3β), phosphorylated, and then ubiquitinated and degraded. When Wnt signaling is activated, β-catenin is stabilized by escaping phosphorylation and enters the nucleus. In the nucleus, β-catenin interacts with the TCF/LEF complex to upregulate the expression of target genes, such as *MYC* and *cyclin D1* [11], which regulate the transition from G1 to the S phase of the cell cycle and are essentially necessary for the proliferation of cells [12]. The wave of Wnt signaling that occurs during early postnatal lung alveologenesis is critical for the final stages of lung development by promoting AT2 cell proliferation [13]. However, increased Wnt/β-catenin activity occurs in patients with BPD [14], and neonatal rodent models of BPD were established [15,16]. The contradiction between the cell proliferation effects of the Wnt pathway and reduced AT2 counts in BPD suggests the existence of proliferation antagonists.

Mammalian Cyclin G1 (CCNG1) are unconventional cyclins, which induce cell proliferation retardation, leading to growth-inhibitory activity by inhibiting different phases of the cell cycle. Studies on Drosophila Cyclin G have shown that Cyclin G negatively regulates cell growth, and overexpression of Cyclin G prevents the G1 to S transition and delays S phase progression [17,18]. High levels of CCNG1 induce a G1-phase arrest [17]. Conversely, lower levels of CCNG1 lack intrinsic growth-inhibitory effects but potentiate alternative reading-frame-mediated growth arrest [19]. In addition, CCNG1 promotes G2/M cell cycle arrest in response to DNA damage and promotes cell recovery after cellular stress [20,21]. Considering that Wnt signaling promotes cell proliferation mainly through the cell cycle, we hypothesize that CCNG1 antagonizes the cell proliferation effects of Wnt signaling in AT2 cells, leading to disrupted alveologenesis. To the best of our current knowledge, no studies have been conducted to explore the relationship between CCNG1 and BPD or Wnt signaling.

In this study, we established an animal model and cell oxidative stress model of BPD to validate the expression alterations of Wnt signaling and CCNG1. Surfactant protein C (SPC), a small-molecule hydrophobic pulmonary surfactant protein secreted by AT2 cells, was used as an AT2 cell marker [22]. Proliferating Cell Nuclear Antigen (PCNA), which functions as an auxiliary protein for DNA polymerase, undergoes a significant increase during cell proliferation, thereby serving as an indicator for assessing the proliferative state of cells [23,24,25]. We therefore utilized these two markers to assess the proliferation of AT2 cells, and our findings revealed that upregulated CCNG1 expression in SPC-expressing cells impairs cell proliferation, possibly by antagonizing the effect of Wnt signaling. This study aimed to elucidate the potential role of CCNG1 in the BPD model, ultimately contributing to a deeper understanding of the pathological mechanisms underlying BPD.

## 2. Materials and Methods

### 2.1. Animal Model of BPD

A total of 4 timed-pregnant C57BL/6 WT mice were obtained from Beijing Vital River Laboratory Animal Technology Co., Ltd. (Beijing, China). An arrest of alveolarization was induced in the mouse pups by exposure to hyperoxia (80% O_2_), as described previously [26]. The timed-pregnant mice were maintained under a 12 h light/12 h dark–light–dark cycle with constant humidity and temperature in the animal care facility. Mouse pups from multiple litters were pooled before being randomly and equally redistributed to two groups (n = 12/group). Beginning on postnatal day 3 (P3), one group was exposed to room air (21% O_2_), and the other group was exposed to hyperoxia (80% O_2_) for 7 days. The dams were rotated between air-exposed and hyperoxia-exposed litters every 24 h. All animal experiments were examined and approved by the Experimental Animal Ethics Committee of Children’s Hospital, Capital Institute of Pediatrics.

### 2.2. Harvesting of Lung Tissues

The mice were euthanized on P10 with intraperitoneal 1% pentobarbital sodium (35 mg/kg). The right ventricle and left ventricle were perfused with ice-cold PBS that had been pressure-inflated at 20 or 25 cmH_2_O, and the lungs were harvested quickly. The lung morphology was observed under a low-power microscope (Axio stemi 508, Carl Zeiss AG, Oberkochen, Germany), and lung lobes were separated. The lower lobe of the right lung was fixed in 4% paraformaldehyde (PFA), and 4 µm thick sections were prepared for hematoxylin and eosin (HE) and immunofluorescence (IF) staining. The remaining lung lobes were preserved at −80 °C for use in Western blotting and RT-qPCR.

### 2.3. HE Staining for Lung Histology

To morphometrically measure lung alveolarization, paraffin-sectioned lung tissues were stained with HE. The radial alveolar count (RAC) was used to evaluate the stage of lung development. Image J software version 1.53a was used to measure the mean alveolar cavity area (MACA).

### 2.4. Western Blot for Protein Expression

Total proteins from the lung tissues and MLE12 cells were lysed using a RIPA buffer (R0020, Beijing Solarbio Science & Technology Co., Ltd., Beijing, China) and 100× Protease Inhibitor Cocktail MIX (IKM1010, Beijing Solarbio Science & Technology Co., Ltd., Beijing, China). The protein concentration was determined by the bicinchoninic acid (BCA; p0010, Beyotime Institute of Biotechnology, Inc., Nantong, China) method. An equal amount of protein (30 µg) of each sample was used for Western blotting. Proteins were then separated by 10% SDS PAGE and transferred onto PVDF membranes. After blocking with 5% non-fat milk for 1 h at 37 °C, the membranes were incubated with the following primary antibodies, diluted in PBS overnight at 4 °C: anti-SPC (10774-1-AP, 1:1000, Proteintech Group, Inc., Wuhan, China, 21 kDa), anti-PCNA (10205-2-AP, 1:1000, Proteintech Group, Inc., Wuhan, China, 29 kDa), anti-CCNG1 (29306-1-AP, 1:200, Proteintech Group, Inc., Wuhan, China, 34 kDa), anti-Wnt5a (55184-1-AP, 1:1000, Proteintech Group, Inc., Wuhan, China, 42 kDa), anti-Wnt3a (sc-74537, 1:500, Santa Cruz Biotechnology Co., Santa Cruz, CA, USA 39 kDa), anti-β catenin (ab78486, 1:1000, Abcam, Cambridge, UK, 92 kDa), anti-β actin (66009-1-Ig, 1:2000, Proteintech Group, Inc., Wuhan, China, 42 kDa), and anti-GAPDH (60004-1-Ig, 1:5000, Proteintech Group, Inc., Wuhan, China, 36 kDa). Following incubation with goat antimouse IgG secondary antibodies (ZB-2305, 1:5000, ZSGB-Bio, Beijing, China) or goat antirabbit IgG secondary antibodies (ZB-2301, 1:5000, ZSGB-Bio, Beijing, China) at 37 °C for 2 h, signals were detected by enhanced chemiluminescence (E002-500mL, Shanghai Seven Sea Biological Medicine Technology Co., Ltd., Shanghai, China). The gray values of the protein bands were analyzed using Image J software version 1.53a.

### 2.5. Reverse Transcription Quantitative PCR (RT qPCR) for mRNA Expression

Total RNA was extracted from the lung tissues or MLE12 using an RNA extraction kit (9767, Takara Bio, Inc., Kyoto, Japan) and was then reverse-transcribed into cDNA using the All-In-One 5X RT MasterMix (G592, Zhenjiang ABM Biotechnology Co., Ltd., Zhenjiang, China). Each targeted cDNA (2 µL) was amplified using the BlasTaq™ 2X qPCR MasterMix (G891, Zhenjiang ABM Biotechnology Co., Ltd., Zhenjiang, China) via the QuantStudio (TM) 7 Flex System, and the primer information is provided in Table S1. To determine the relative gene expression, the target mRNA expression was calculated relative to β-actin using the 2^−ΔΔCt^ method.

### 2.6. IF Staining for Protein Expression and Colocalization

Before IF staining, the lung sections or MLE12 cells were fixed with 4% PFA (P110, Beijing Solarbio Science & Technology Co., Ltd., Beijing, China) for 15 min. After blocking with 5% goat serum (SL038, Beijing Solarbio Science & Technology Co., Ltd., Beijing, China) at room temperature for 60 min, they were incubated with combinations of 1 or 2 primary antibodies overnight at 4 °C as follows: rabbit polyclonal anti-SPC (10774-1-AP, 1:200, Proteintech Group, Inc., Wuhan, China) and mouse monoclonal anti-AQP5 (sc-514022, 1:50, Santa Cruz Biotechnology Co., CA, USA ); rabbit polyclonal anti-SPC and mouse monoclonal anti-Wnt3a (sc-74537, 1:50, Santa Cruz Biotechnology Co., CA, USA ); rabbit polyclonal anti-SPC and mouse polyclonal anti-CCNG1 (SAB1405572, 1:100, sigma, St. Louis, MO, USA); rabbit polyclonal anti-CCNG1 (29306-1-AP, 1:200, Proteintech Group, Inc., Wuhan, China) and mouse monoclonal anti-Wnt3a (sc-74537, 1:50, Santa Cruz Biotechnology Co., CA, USA ); or rabbit polyclonal anti-SPC (10774-1-AP, 1:200, Proteintech Group, Inc.). The sections were then incubated with goat antirabbit IgG (SA00013-4, 1:200, Proteintech Group, Inc., Wuhan, China) and goat antimouse IgG (SA00013-1, 1:200, Proteintech Group, Inc., Wuhan, China) secondary antibodies, coupled with fluorescein isothiocyanate, in the dark for 1 h at room temperature, followed by 4′,6 diamidino 2 phenylindole (DAPI, D820010, Beijing Solarbio Science & Technology Co., Ltd., Beijing, China) for nuclear staining for 10 min at room temperature. Double IF images were acquired using a confocal laser scanning microscope (DMi8, Leica, Wetzlar, Germany ), and 6 fields of view were randomly selected for each sample.

### 2.7. Transcriptomics Study

The data of scRNA-seq were acquired from NCBI GEO: GSE211356, and bulk RNA-seq data were acquired from NCBI GEO: GSE216046. Seurat objects were created using RStudio (v4.4.1), and then, data were merged. The methods for data processing, quality control, integration, and cluster annotation refer to a previous study [27]. The expression matrices for air and hyperoxia were loaded into R as Seurat objects, retaining only cells in which more than 200 genes were detected. SCTransform was used to normalize each sample and select highly variable genes. PCA was then run on the top 3,000 variable genes, and the data were then clustered at a low resolution (dims = 1:30; resolution = 0.3) with the Louvain algorithm implemented in the FindClusters function in Seurat. UMAP embeddings were generated on the top 30 PCs. To identify major cell type subsets (epithelial, endothelial, immune, mesenchymal), we assessed the expressions of the canonical markers Epcam, Cldn5, Ptprc, and Col1a2. We also assessed additional genes discriminating these clusters by performing a simple Wilcoxon rank-sum test with the FindAllMarkers function in Seurat. The bulk RNA-seq data for genes were analyzed in R using the DESeq2 software package (version 1.30.1). The volcano plot showed the upregulated and downregulated transcript levels of DEGs, with a *p*-value < 0.05 and |log2FoldChange| ≥ 1. The signaling pathways were identified by KEGG pathway enrichment analysis. All terms shown are significantly enriched (*p*-value < 0.05).

### 2.8. Cells Model of BPD

Mouse alveolar epithelial cells (MLE12, Hunan Fenghui Biotechnology Co., Ltd., Changsha, China) were used to construct an in vitro model of oxidative stress, as described in a previous study [28,29]. The siRNAs (CCNG1 (mouse) si-RNA) and corresponding negative controls (si-nc negative control) of CCNG1 were obtained from Sangon Biotech. MLE12 were plated in a cell dish (Corning, NY, USA) with DMEM/F12 (D6570, Solarbio, Beijing, China) containing 10%FBS (S9020, Solarbio, Beijing, China) at the density of 10,000 cells/well. After 12 h (on day 1), the medium of the air group and H_2_O_2_ group were supplemented with RFect siRNA/miRNA Transfection Reagent (Changzhou Bio-generating Biotechnology Co., Changzhou, China) containing si-nc, and the medium of the Si-CCNG1 + H_2_O_2_ group was supplemented with RFect siRNA/miRNA Transfection Reagent containing si-CCNG1. Subsequently, on day 3, the medium was changed to DMEM/F12 with 10%FBS. Twenty-four hours later (on day 4), the medium of the H_2_O_2_ group and Si-CCNG1 + H_2_O_2_ group was supplemented with the stimuli hydrogen peroxide (H_2_O_2_) at 100 μM as a prooxidative agent. After an additional 12 h, the cells were harvested for further analysis. The sequence of the si-CCNG1 duplex is shown in Table S2.

### 2.9. Statistical Analysis

GraphPad prism (9.5) was used to analyze the data, which are presented as the mean ± standard deviation (SD). The comparisons of two group samples were analyzed with the *t*-test, and multiple comparisons were performed using one-way ANOVA with Bonferroni’s post hoc test. *p* < 0.05 was considered to indicate a statistically significant difference.

## 3. Results

### 3.1. Hyperoxia Exposure Led to Growth Retardation and a Decrease in the Population of AT2 Cells

**Figure 1:**
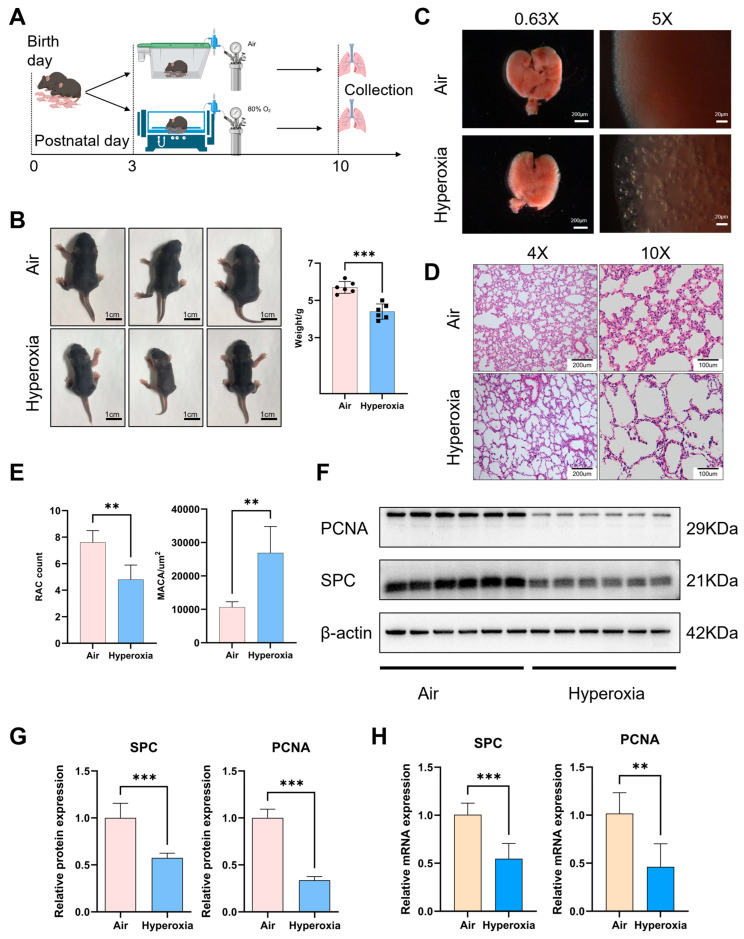

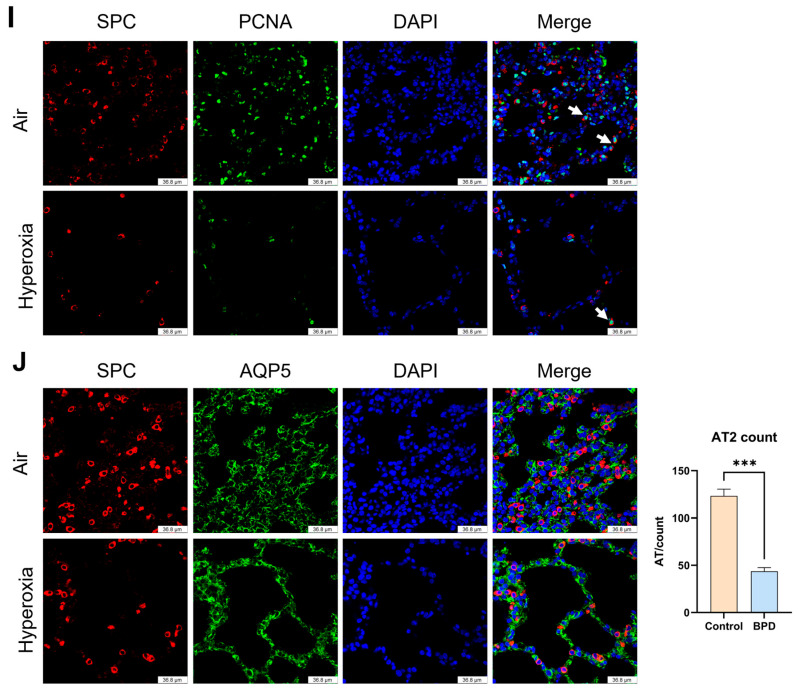
Hyperoxia disrupted alveologenesis and led to a decrease in the population of AT2 cells. (**A**) Schematic diagram of the hyperoxia model of BPD in neonatal mice. Mouse pups were exposed to room air (21% O_2_) or hyperoxia (80% O_2_) from the postnatal days (P)3 to P10. On P10, lungs were harvested for subsequent detection. (**B**) Representative images of the body size. The weight of the hyperoxia group mice decreased (n = 6). (**C**) The morphology of hyperoxia group lungs exhibited vacuole-like morphological changes (left panel: 0.63×, scale bar = 200 μm; right panel: 5×, scale bar = 20 μm). (**D**) Representative images of HE-stained lungs (left panel: scale bar = 200 μm; right panel: scale bar = 100 μm). (**E**) Quantification of the radial alveolar count (RAC) and mean alveolar cavity area (MACA) (n = 5). (**F**,**G**) Western blot analysis of SPC, PCNA (n = 6). (**H**) The detection of SPC, PCNA mRNA expression (n = 6). (**I**) Representative images of IF staining for SPC (red) and PCNA (green) (scale bar = 36.8 μm). (**J**) Representative images of IF staining for SPC (red) and AQP5 (green) (scale bar = 36.8 μm). The count of AT2 cells (n = 6). Data are shown as the means ± SD; ** *p* < 0.01; *** *p* < 0.001. PCNA: proliferating cell nuclear antigen. SPC: surfactant protein C. AQP5: Aquaporin 5. DAPI: 4′,6-diamidino-2-phenylindole.

The schematic diagram of the BPD model construction is shown in Figure 1A. Compared to the air group, the hyperoxia group exhibited severe growth retardation and a notable decrease in body weight (Figure 1B). Under low magnification (0.63× and 5×), the lungs of the hyperoxia group exhibited vacuole-like morphological changes (Figure 1C), and a histological examination using HE staining revealed significant alterations in the alveolar structure (Figure 1D). Specifically, alveolar simplification was evident, characterized by enlarged alveolar spaces, a substantial increase in the MACA, a decrease in the number of alveoli per unit area, and a marked reduction in the RAC (Figure 1E). These results indicate that the prominent feature of BPD that is caused by hyperoxia is alveolar retardation, confirming the successful establishment of our hyperoxia model of BPD.

The expression of PCNA decreased, indicating that the overall proliferative ability of the lungs was reduced in hyperoxia conditions (Figure 1F–I). And the number of AT2 cells expressing PCNA in lung tissue was reduced, suggesting that most AT2 cells are not in a proliferative state (Figure 1I). The expression of the AT2 cell marker SPC in the lungs of the hyperoxia group was also obviously lower than that in the air group (Figure 1F,G). IF staining also showed a downregulated expression of SPC, and quantification confirmed a marked reduction in the number of AT2 cells in the hyperoxia group (Figure 1J). These results indicate that hyperoxia leads to a reduction in AT2 cells, which underlies the disruption of alveologenesis and leads to the pathogenesis of BPD.

### 3.2. The Expression Level of CCNG1 Increases Under Hyperoxia in RNA-Seq Analysis

**Figure 2:**
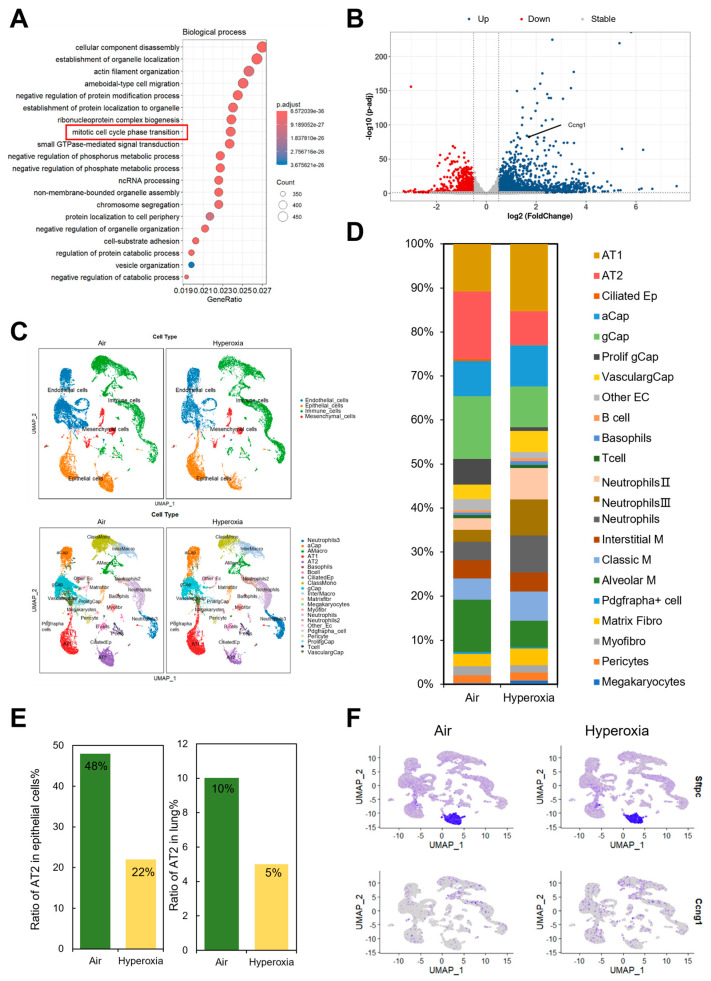
RNA-seq and scRNA−seq analysis of the air and hyperoxia group mice. (**A**) Hyperoxia−impacted signaling pathways as identified by GO analysis. (**B**) Volcano plot showing the upregulated transcript levels of *CCNG1* of differentially expressed genes. (**C**) UMAP plot of all scRNA−seq data, showing a total of 4 major cell groups (immune cells, endothelial cells, epithelial cells, and mesenchymal cells) and 22 cell clusters. Cell populations are colored as indicated by the legend. (**D**) The compositions of the 22 cell clusters are colored as indicated by the legend in the air and hyperoxia group. (**E**) The relative proportion of AT2 cells from epithelial cells and all lung cells. (**F**) Feature plots showing the expression of *SPC* and *CCNG1*.

The results of the gene ontology (GO) analysis showed that mitotic cell cycle phase transition was one of the enriched pathways (Figure 2A). The volcano plot revealed the upregulated transcript levels of *CCNG1* of differentially expressed genes (Figure 2B). These results indicate that the reduction in AT2 cells may be correlated with the increased expression of *CCNG1*. We analyzed the scRNA-seq data to identify four major cell lineages (endothelial cells, epithelial cells, immune cells, and mesenchymal cells) and further categorized these major cell lineages into 22 sub-clusters (Figure 2C). When comparing the lung tissues from the air group to the hyperoxia group, we observed a pronounced decrease in the frequency of AT2 cells (Figure 2D,E), which was consistent with the results obtained from our mouse model. The feature plots further confirmed that the expression of *CCNG1* was upregulated in the hyperoxia group (Figure 2F).

### 3.3. The Elevated Expression of CCNG1 Is Accompanied by the Upregulation of the Wnt Signaling

**Figure 3:**
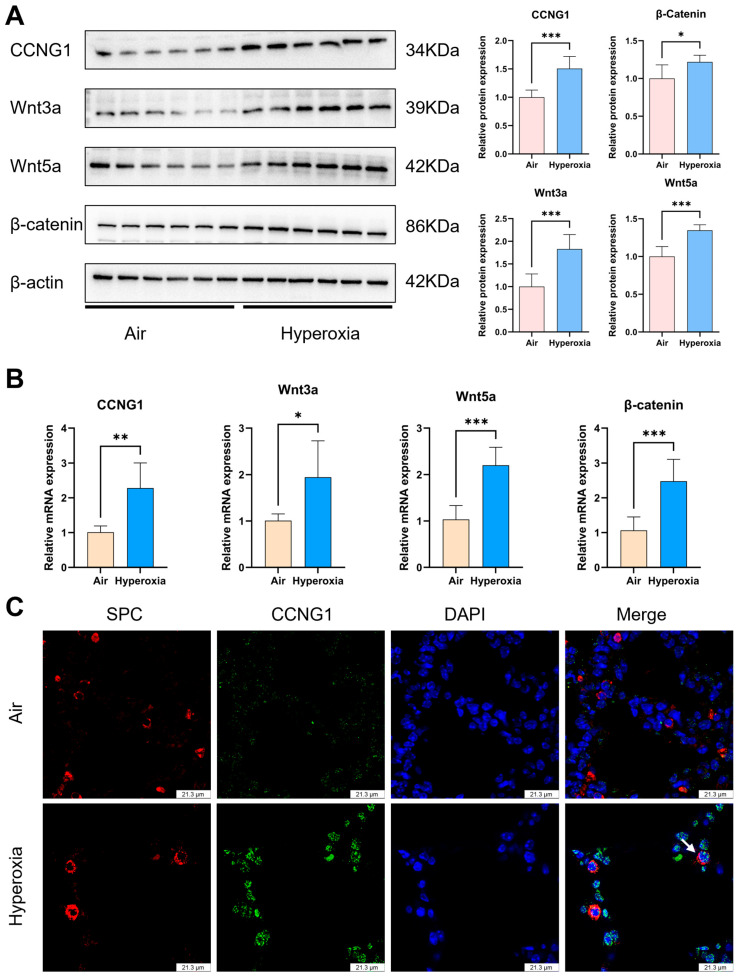

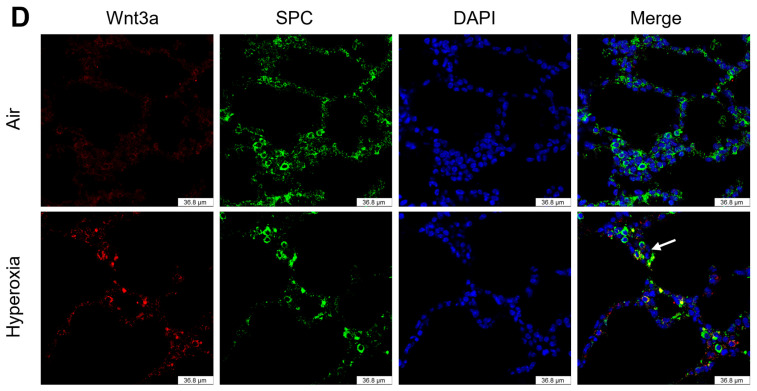
In the hyperoxia group, upregulated CCNG1 in AT2 cells was accompanied by increased expressions of Wnt3a, Wnt5a, and β−catenin. (**A**,**B**) Western blot analysis and RT-qPCR verification of CCNG1, Wnt3a, Wnt5a, and β−catenin expressions (n = 3). (**C**) Representative images of IF staining for SPC (red) and CCNG1 (green), and the white arrow shows the colocalization of SPC and CCNG1 (scale bar = 21.3 μm). (**D**) Representative images of IF staining for Wnt3a (red) and SPC (green). The white arrow shows the colocalization of SPC and Wnt3a (scale bar = 36.8 μm). Data are shown as the means ± SD; * *p* < 0.05; ** *p* < 0.01; *** *p* < 0.001. SPC: surfactant protein C. CCNG1: cyclin G1. DAPI: 4′,6−diamidino−2−phenylindole.

In the hyperoxia group lungs, both the mRNA and protein expression levels of CCNG1 expression increased (Figure 3A,B). And the relationship between AT2 cells and upregulated CCNG1 was studied by IF staining, and the results showed that the AT2 cell markers SPC and CCNG1 were colocalized in the hyperoxia group (Figure 3C). Those results suggested that hyperoxia-induced upregulation of CCNG1 is associated with a reduced count of AT2 cells. The protein and mRNA expression levels of Wnt3a, Wnt5a, and β-catenin increased in the hyperoxia group (Figure 3A,B). The result of IF staining revealed that the Wnt3a can be colocalized with SPC in the hyperoxia group (Figure 3D), indicating that the proliferation effects of Wnt signaling in AT2 cells are weakened after hyperoxia exposure related to a high expression of CCNG1.

### 3.4. Inhibition of CCNG1 in MLE12 Cells Promotes Cell Proliferation

**Figure 4:**
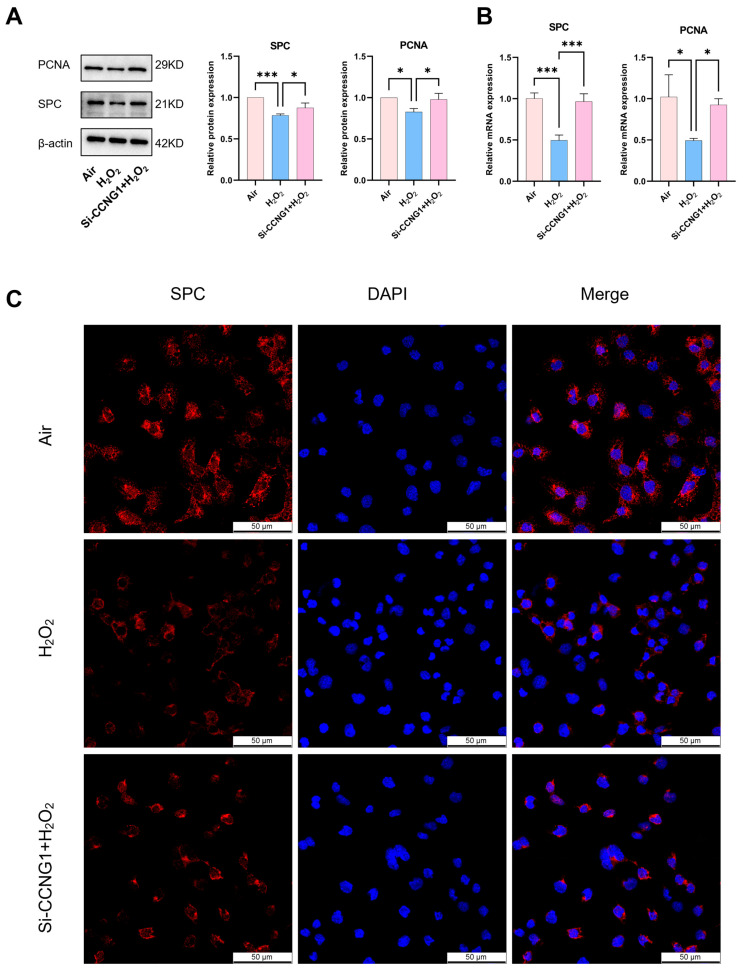
Inhibition of CCNG1 in MLE12 cells promotes cell proliferation in the oxidative stress model. (**A**) The protein expressions of SPC and PCNA in the air, H_2_O_2_, and Si−CCNG1 + H_2_O_2_ groups (n = 3). (**B**) The mRNA expressions of SPC and PCNA in the three groups (n = 3). (**C**) Representative images of IF staining for SPC (red) (scale bar = 50 μm). Data are shown as the means ± SD; * *p* < 0.05; *** *p* < 0.001.

The MLE12 cell line was isolated and cultured from lung tumor cells and retained characteristics of respiratory epithelial cells [30]. To further explore the implications of CCNG1 on the cellular proliferation of AT2 cells, we used MLE12 to construct the oxidative stress model and siRNA to knock down the *CCNG1* expression (Supplementary Figure S1). Compared with the air group, the expression of the cell proliferation marker PCNA and SPC expression were decreased in the H_2_O_2_ group, while the proliferation ability was partially restored after interference with CCNG1 (Figure 4A,B). IF staining provided additional insights, revealing a decrease in SPC expression in the H_2_O_2_ group (Figure 4C). After CCNG1 was inhibited, SPC expression was partially restored.

### 3.5. Inhibition of CCNG1 Regresses Wnt Signaling

**Figure 5:**
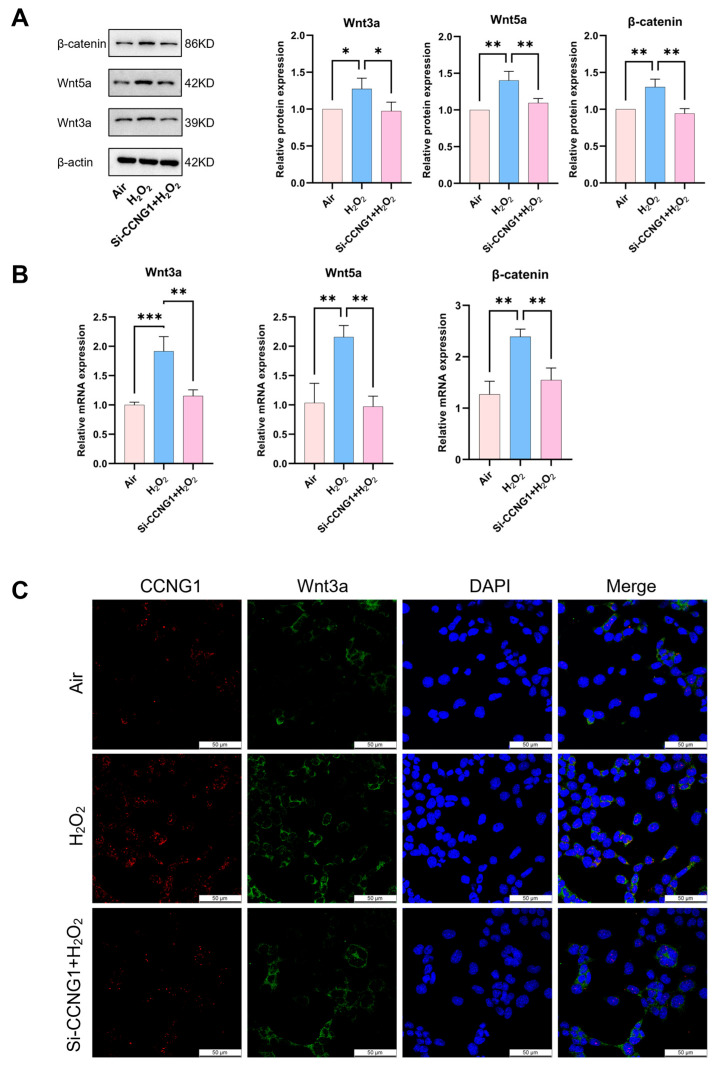
The inhibition of CCNG1 reverted the expression of Wnt signaling in an oxidative stress model. (**A**) The protein expressions of Wnt3a, Wnt5a, and β−catenin in the air, H_2_O_2_, and Si−CCNG1 + H_2_O_2_ groups (n = 3). (**B**) The mRNA expressions of Wnt3a, Wnt5a, and β−catenin in the three groups (n = 3). (**C**) Representative images of IF staining for CCNG1 (red) and Wnt3a (green) (scale bar = 50 μm). Data are shown as the means ± SD; * *p* < 0.05; ** *p* < 0.01; *** *p* < 0.001.

The protein and mRNA expression levels of Wnt3a, β−catenin, and Wnt5a increased in the H_2_O_2_ group (Figure 5A,B). When CCNG1 was inhibited, Wnt3a, β−catenin, and Wnt5a expressions almost reverted to the air group levels (Figure 5A,B). The IF staining results also showed increases in Wnt3a in the H_2_O_2_ group. Heightened Wnt3a expression correlated with elevated CCNG1 levels (Figure 5C). After CCNG1 was inhibited, the Wnt3a expressions were decreased. The decrease in Wnt3a expression after inhibiting CCNG1 suggests that CCNG1 might function as a negative regulator to impede the Wnt signaling−mediated cell proliferation, thereby contributing to the decreased number of AT2 cells in the BPD model.

## 4. Discussion

Alveolar simplification is the primary pathological alterations observed in BPD [31]. The alveolar simplification of BPD is primarily associated with two types of alveolar epithelial cells: AT1 and AT2. AT1 cells are thin squamous cells that intimate contact with the endothelial cells lining the capillaries in the lungs, forming a gas exchange area [32]. AT2 cells serve as progenitor cells for ATI cells, providing a source of cellular repair after lung injury [33,34]. AT2 cells proliferate to replace the lost cells, and once sufficient cell numbers are restored, some differentiate into ATI cells to restore the alveolar structure. AT2 injury is considered the key mechanism in BPD−related pulmonary epithelial injury [35]. Inadequate repair of lung tissue following hyperoxia−induced injury is significantly attributed to the dysregulated proliferation and differentiation of AT2 cells [36,37]. Signaling pathways that promote AT2 cell proliferation have been identified, including epidermal growth factor (EGF), Wnt/β−catenin, and others [38,39,40]. Although we have some understanding of the mechanisms that drive AT2 cell proliferation, the signaling pathways that retard AT2 cell proliferation have remained elusive. In this study, we observed that CCNG1, a key negative regulator of the cell cycle, was robustly upregulated in AT2 cells. Notably, upon perturbing CCNG1 expression, cell proliferation was restored, which indicated that CCNG1 might be involved in the retardation of AT2 cell proliferation in the BPD model. This finding contributes to an enhanced comprehension of the pathogenesis of BPD.

Recently, research has shown that CCNG1 overexpression is heavily related to stem and malignant cell proliferation [41,42]. However, other studies found that CCNG1 actively participates in the intricate process of cell mitosis, exerting a negative regulatory influence on the rate of cellular proliferation [17,43], by regulating both the G1/S and G2/M phases of the cell cycle [20,44]. CCNG1 expression increased in Ishikawa and HEC−1−B cells after treatment with progesterone, and the cell proliferation was suppressed [45]. In lungs, miR−23b inhibited cancer cell proliferation by directly targeting CCNG1 [46]. These studies suggested that CCNG1 may act differently in different stem cells or tumor cells. To the best of our knowledge, the role of CCNG1 remains elusive in neonatal lungs and AT2 cells. Our results indicated that CCNG1 was significantly upregulated and colocalized with SPC in the hyperoxia group. And cell proliferation ability was restored when CCNG1 expression was perturbed. Those results demonstrated that CCNG1 can regulate the proliferation of AT2 cells in BPD. This suppression of cell proliferation may contribute to the homeostatic regulation of AT2 cells in response to acute injury. In the neonatal lung, exposure to hyperoxia leads to the formation of ROS. Previous studies have revealed that oxidative DNA damage occurred in AT2 during early−stage BPD, as confirmed by in vitro and in vivo hyperoxia exposure experiments [47], and oxidative DNA damage was the crucial mechanism in the pathogenesis of BPD [48]. In response to oxidative DNA damage, CCNG1 exerts an inhibitory effect on cell proliferation, and the maintenance of proper cell cycle arrest is essential for cell survival [49]. Our research has identified a novel function of CCNG1, namely its role in maintaining cellular homeostasis through the inhibition of AT2 cell proliferation in BPD. Based on our results showing that the Wnt pathway is highly expressed in AT2 cells, we hypothesize that CCNG1 acts as an antagonist, counteracting the proliferative effects mediated by Wnt signaling.

The canonical Wnt signaling promotes cell proliferation by triggering target genes, including cyclin D1 and c−myc, which regulate the transition from the G1 to the S phase of the cell cycle [12]. The inhibition of Wnt/β−catenin signaling has been shown to impede AT2 cell proliferation during the regenerative process following lung injury [38]. Furthermore, studies using lineage tracing and inducible AT2 cell−specific gene−deficient mice have confirmed that Wnt/β−catenin signaling is critical for AT2 cell proliferation after lung injury [33,39]. Interestingly, Wnt signaling is required not only for AT2 cell proliferation but also for the maintenance of the phenotype. Wnt signaling is important for the maintenance of the stemness of these Axin2^+^ AT2 cells [39]. During alveoli regeneration after acute lung injury, active Wnt signaling is important for the expansion of the Axin2^+^ AT2 cells and the inhibition of differentiation of AT2 to AT1 [13,33,39].

However, other studies found that both canonical and non−canonical Wnt signaling pathways have been shown to regulate AT2 cell differentiation [50]. Both canonical Wnt3a and non−canonical Wnt5a ligands are derived from the neighboring LGR5^+^ stromal cells to promote the alveolar differentiation of both club and AT2 cells [51]. It is not yet determined why these contrasting results exist. Our findings revealed a notable upregulation of Wnt3a, β−catenin, and Wnt5a in AT2 cells. Consistent with the trend of our results, the finding of human lung samples and murine models of human BPD show that IF colocalization demonstrated a consistent pattern of elevated nuclear−phosphorylated β−catenin in the lung epithelium and surrounding mesenchyme in BPD [52]. The upregulation of the Wnt pathway could not increase the number of AT2 cells that are exposed to hyperoxia, which further suggested the existence of a proliferation antagonist.

Our findings revealed a significant colocalization of elevated CCNG1 and Wnt3a within AT2 cells. When CCNG1 expression was perturbed, the cell proliferation ability was restored, and the expression of Wnt signaling was also significantly decreased. Consequently, when the cell proliferation returns to normal, the Wnt signaling no longer needs to be overexpressed, which makes AT2 cells shift from proliferation to differentiation into AT1 cells [13]. The inhibitory effect of CCNG1 on Wnt signaling may occur in the cell cycle. Previous studies have shown that Wnt signaling promotes cell proliferation by regulating the progression of the G1 to S phase [12], and the target protein of Wnt signaling, c−myc, promotes the S/G2 and G2/M phases of the cell cycle [53]. However, CCNG1 prolonged the duration of the S phase and arrested G2/M phases [17,20,44]. Therefore, when CCNG1 is upregulated, cell mitosis stops leading to proliferation arrest. However, the specific targeted sites of CCNG1 to antagonize the effects of Wnt signaling in AT2 cells still need to be further explored.

Our study serves as a preliminary investigation into the role of cyclin in the proliferation of AT2 cells. However, it is noteworthy that a limitation of this research is the lack of identification of the specific binding site between CCNG1 and the Wnt pathway. Furthermore, the specific cell cycle phase that is disrupted in AT2 cells within the hyperoxia−induced BPD model remains unclear. Whether the lung morphometry of pups exposed to hyperoxia can undergo improvement following the intervention of CCNG1 inhibition (such as DeltaRex−G), along with the methodologies to enhance AT2 cell proliferation and mitigate AT2 cell loss in the lungs, continues to merit further investigation. In the future, we aim to delve deeper into these questions by generating *CCNG1*^−/−^ mice. It is necessary to establish a hyperoxia−induced BPD model using primary mouse alveolar epithelial cells and *CCNG1*^−/−^ mice. And the application of transcriptomics, proteomics, and flow cytometry to assess cell cycle dynamics is useful to explore the effect of the Wnt pathway and CCNG1 in AT2 cell proliferation. Additionally, the ultimate goal is to effectively assess the feasibility of translating these theoretical findings into clinical applications.

## 5. Conclusions

In conclusion, our findings underscore the pivotal role of a marked reduction in the AT2 cell population as the primary culprit behind disrupted alveologenesis in BPD neonatal lungs. Our results demonstrate that the CCNG1 expression was increased in AT2 cells of BPD, and the higher CCNG1 impaired, at least in part, the proliferation ability and contributed to BPD by the antagonistic effects on Wnt signaling in AT2 cells. As far as we know, there is no study that explores the role of CCNG1 in BPD. To unravel the intricate functions of CCNG1 further, we aspire to embark on additional studies that delve into its role in modulating the downstream components of Wnt signaling during the intricate orchestration of the cell cycle.

## Data Availability

The data that support the findings of this study are available from the corresponding author.

## Supplementary Materials

The following supporting information can be downloaded at https://www.mdpi.com/article/10.3390/biom15010101/s1, Figure S1: Original Images for Blots in Figure 1; Figure S2: Original Images for Blots in Figure 3; Figure S3: Original Images for Blots in Figure 4; Figure S4: Original Images for Blots in Figure 5; Table S1: Sequence of primer; Table S2: Sequence of Si−CCNG1.
