# Supplementary material for: Cyclin G1 Regulates the Alveolarization in Models of Bronchopulmonary Dysplasia by Inhibiting AT2 Cell Proliferation
Source: Biomolecules. 2025 Jan 10;15(1):101. doi: 10.3390/biom15010101 (PMC11764269; doi:10.3390/biom15010101)
Supplement: Supplementary file 1 [file biomolecules-15-00101-s001.zip › biomolecules-3285469-WB images.pdf]

Original Images for Blots in Figure

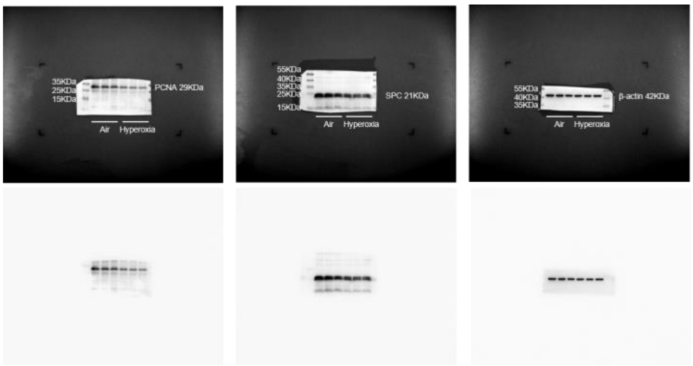

Figure S1. Original Images for Blots in Figure 1.

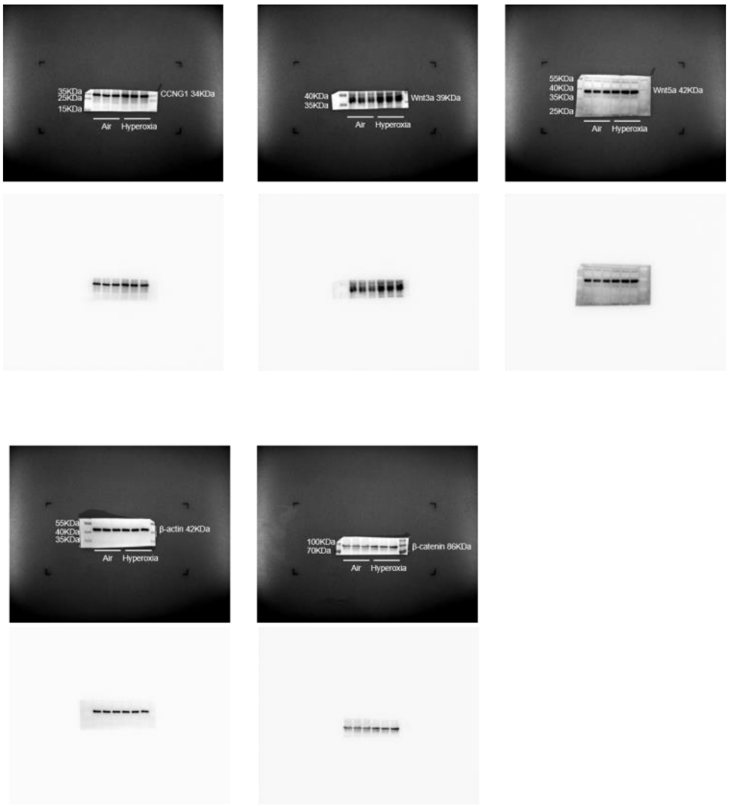

Figure S2. Original Images for Blots in Figure 3.

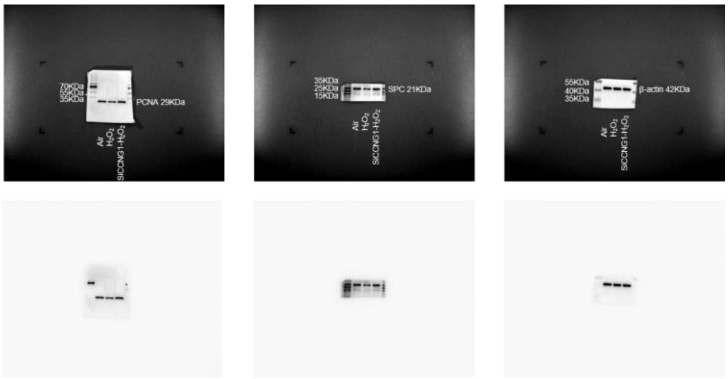

Figure S3. Original Images for Blots in Figure 4.

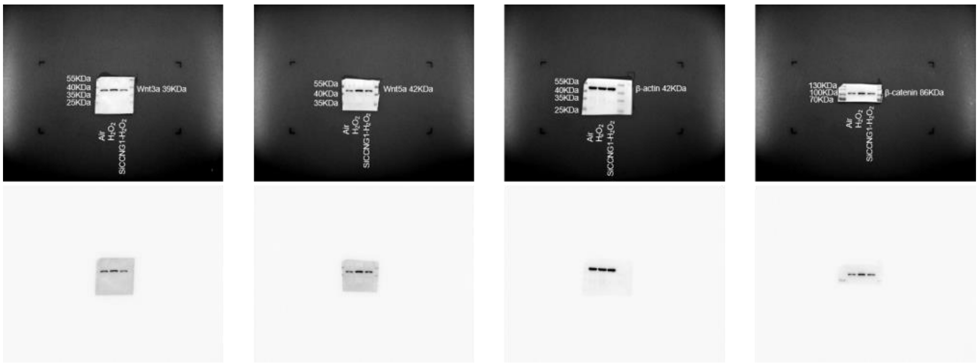

Figure S4. Original Images for Blots in Figure 5.
